# Biogenic nanoparticles from bacteria: a perspective on integrated applications in sustainable agriculture

**DOI:** 10.3389/fmicb.2025.1724288

**Published:** 2025-12-02

**Authors:** Natalia Bilesky-Jose, Renata Lima

**Affiliations:** Laboratory for Evaluation of the Bioactivity and Toxicology of Nanomaterials, University of Sorocaba (UNISO), Sorocaba, São Paulo, Brazil

**Keywords:** green nanotechnology, bacterial synthesis, sustainable agriculture, nanofertilizers, phytosanitary protection

## Abstract

The growing demand for sustainable agricultural solutions has driven the development of technologies that contribute to modern agriculture, which aims to achieve greater productivity while maintaining environmental responsibility. Biogenic nanoparticles (BNPs) synthesized using bacteria are emerging as a promising alternative to conventional methods, offering a green approach for producing nanomaterials with agricultural applications. This Perspective highlights the mechanistic basis of bacterial nanoparticle biosynthesis and strategies for genetic and metabolic optimization to enhance yield and functionality, accentuating their potential applications as phytosanitary agents and controlled-release fertilizers. We further propose an integrative “BNP–Plant–Microbiome” framework, in which microbial consortia and multi-nanoparticle formulations could synergistically deliver nutrients, boost stress resilience, and suppress pathogens. Future progress will depend on addressing key challenges in biosafety, regulatory compliance, and large-scale bioprocessing, as well as integrating BNPs with precision agriculture and data-driven monitoring tools. Ultimately, bacterial BNPs have the potential to redefine agricultural sustainability by coupling microbial innovation with circular, resource-efficient crop management systems.

## 1 Introduction

Modern agriculture faces the challenge of increasing food production for an estimated population of 9.7 billion by 2050. In addition, concerns exist about reducing the environmental impact of conventional practices (United Nations, 2019; FAO, 2021). Traditional methods based on synthetic agrochemicals are limited in terms of efficiency and in their contribution to environmental contamination. Additionally, agrochemicals persist in the environment and can lead to pathogen resistance (Qiu et al., 2022).

Nanotechnology offers innovative solutions by manipulating materials with unique properties, including a high surface-to-volume ratio, increased reactivity, and cell penetration capacity, making them suitable for more efficient agricultural applications (Bouhadi et al., 2025).

Among various production methods, biogenic synthesis has gained attention owing to its environmental and economic advantages. This synthesis represents a green alternative to physicochemical methods, which often involve extreme conditions, high energy consumption, and toxic reagents (Arora A. et al., 2024), with the combination of nanotechnology and natural processes capable of solving problems such as high costs, toxicity, instability, and chemical waste generation associated with physicochemical methods (Rani et al., 2021). This approach not only reduces the environmental impact but also increases the biocompatibility and biodegradability of nanoparticles, making them suitable for a wide range of applications, including agriculture and environmental remediation (Guilger-Casagrande and Lima, 2019; Khan et al., 2022; Mgadi et al., 2024). Overall, biogenic synthesis is a promising alternative to traditional methods and aligns with the principles of green chemistry and sustainable development (Álvarez-Chimal and Ángel Arenas-Alatorre, 2023; Tsekhmistrenko et al., 2020).

The use of bacteria for the synthesis of biogenic nanoparticles (BNPs) is an interesting approach, as they possess unique metabolic capabilities for the controlled reduction of metal ions under mild conditions, resulting in nanoparticles with well-defined properties (Verma et al., 2024), such as controlled morphology and size and natural stabilization through biomolecules, and facilitating large-scale production using established fermentation processes (Saxena et al., 2025).

**Figure 1:**
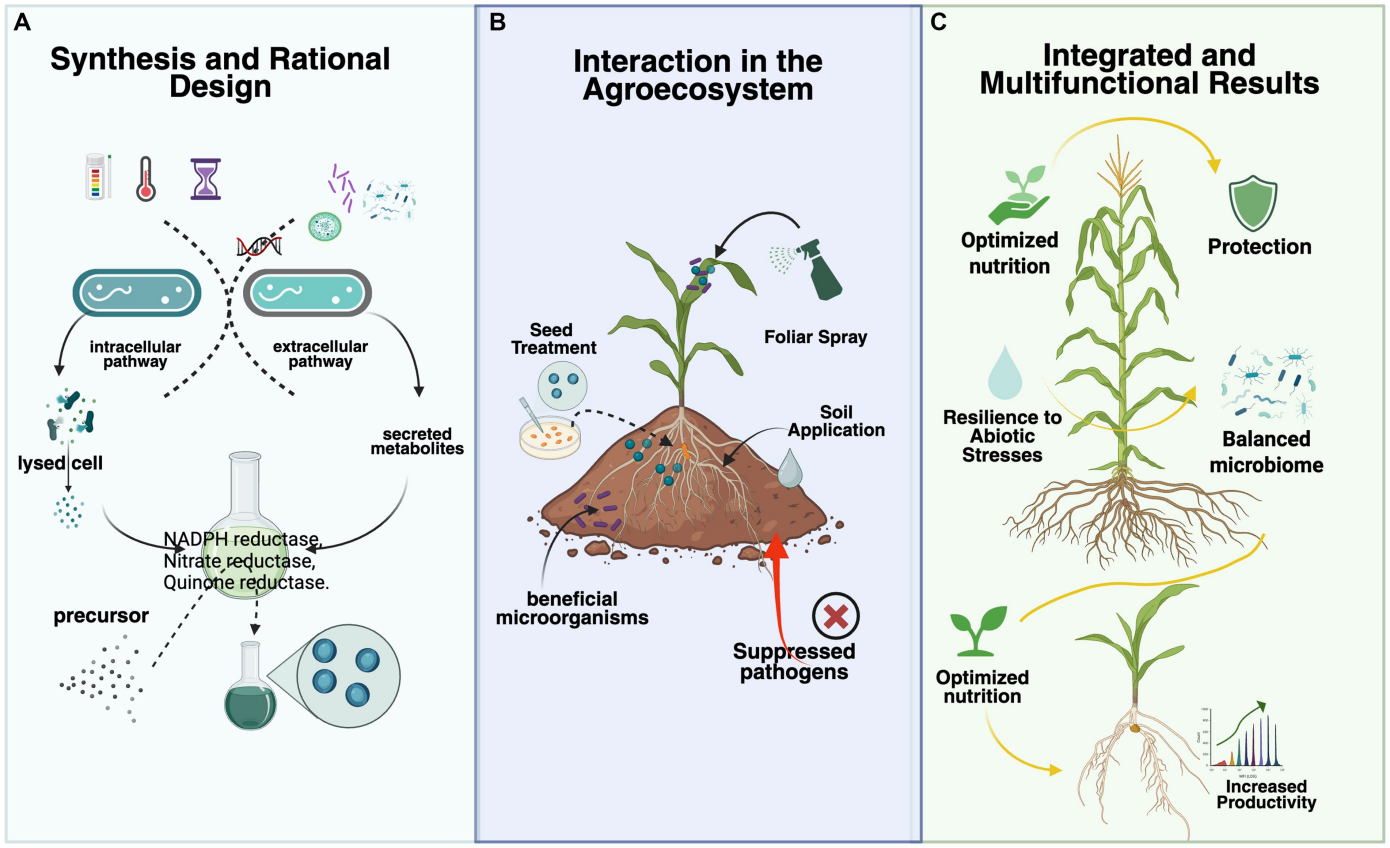
This framework illustrates the three key stages of bacterial biogenic nanoparticle (BNP) technology for sustainable agriculture. **(A)** Rational design and synthesis: bacterial cells produce BNPs through intracellular and extracellular pathways, which can be modulated by adjusting culture parameters and selecting specific strains to obtain nanoparticles with desired properties. **(B)** Application and interaction: BNPs are applied to the agroecosystem via seeds, foliage, or soil, where they interact with the plant and its associated microbiome, selectively modulating microbial communities and delivering nutrients and bioactive compounds. **(C)** Integrated outcomes: the synergistic interaction among BNPs, the plant, and the microbiome results in multifunctional benefits, including enhanced nutrition, pathogen protection, stress resilience, and a balanced microbiome, leading to increased productivity.

In an agricultural context, bacterial biogenic nanoparticles have the potential to provide phytosanitary protection through their intrinsic antimicrobial properties and controlled nutrient-release systems, such as in nanofertilizers (Figure 1). This dual functionality represents a unique opportunity for integrated agricultural solutions that simultaneously address plant nutrition and pathogen protection while reducing environmental impact (Rupanshi et al., 2025).

In this perspective, we argue that the next breakthrough in the application of bacterial BNP to sustainable agriculture extends beyond their isolated functions as biofertilizers or biopesticides. We indicate that the true transformative potential of these nanoparticles lies in their capacity to act as modulators of integrated systems, in which rationally designed, synergistic interactions among the BNP, the host plant, and its associated microbiome result in a more resilient, productive, and ecologically balanced agricultural ecosystem.

We propose an innovative conceptual framework for this field that addresses the biological complexity and “synergy” of BNP. Possibilities such as the use of genetic engineering in bacteria to improve nanoparticle production, the reuse of agricultural waste, and the strategic combination of different BNPs with current technologies are also discussed.

## 2 Mechanisms of bacterial biosynthesis of nanoparticles

The ability of bacteria to synthesize metallic nanoparticles is based on resistance mechanisms that evolved to mitigate heavy metal toxicity. These mechanisms involve specialized enzyme systems that reduce metal ions to elemental forms, resulting in the precipitation of nanoparticles inside or outside the cells (Iravani et al., 2014; Shaikh et al., 2024; Tsekhmistrenko et al., 2020).

The synthesis process is divided into three stages: (I) reduction, (II) nucleation and growth, and (III) stabilization. The efficiency and characteristics of the nanoparticles depend on the metal ion concentration, pH, temperature, incubation time, and bacterial cell density (Guilger-Casagrande and Lima, 2019; Lengke et al., 2006).

The precise mechanism of reduction in biosynthesis has not yet been fully established; however, studies in plants have attributed this action to the activities of polyphenols (flavonoids, phenolic acids, and lignans), terpenoids, carotenoids, and sterols (Ankamwar et al., 2020). In fungi and bacteria, the cofactor nicotinamide adenine dinucleotide phosphate (NADPH) and enzyme nitrate reductase are often responsible for reducing metal ions to nanoparticles, with NADPH also acting independently (Hietzschold et al., 2019). Other bacterial metabolites, such as proteins containing sulfur, arginine, aspartic acid, cysteine, glutamic acid, lysine, and methionine, may also contribute to this reduction (Rai et al., 2021).

The NADH-dependent reductase mechanism involves the oxidation of NADH to NAD+, which releases electrons that are transferred to metal ions via an electron transport chain. This process reduces metal ions to their elemental state (M^0^), leading to the formation of metal nuclei that subsequently grow into stable nanoparticles (Bhatnagar and Aoyagi, 2025; Mikhailova, 2024; Pan et al., 2025).

Quinone reductase facilitates electron transfer processes that are essential for the reduction of metal ions and plays an important role in the formation of iron and copper nanoparticles (Gahlawat and Choudhury, 2019; Sidhu et al., 2025; Yadav et al., 2023). In addition to stabilizing the reduced metal ions, preventing their reoxidation, and promoting the formation of stable nanoparticles (Durán et al., 2005), quinone reductase can operate under moderate conditions, which is an advantage in the synthesis process (Zou et al., 2021).

Another important component is microbial biosurfactants, whose amphiphilic nature allows them to stabilize nanoparticles by preventing aggregation and promoting the formation of stable colloidal structures, making them effective for green synthesis processes (Carmona et al., 2023; Sadiq et al., 2022). Bacterial exopolysaccharides (EPSs) also play a significant role in this context, as they can reduce metal ions and stabilize nanoparticles, thereby increasing their biocompatibility (Escárcega-González et al., 2018).

Bacterial synthesis enables control of nanoparticle morphology and size by modulating cultivation conditions and enzyme expression (Lahiri et al., 2021). Proteins and polysaccharides in the bacterial cell wall act as natural stabilizing agents, preventing aggregation and conferring colloidal stability. This biological stabilization offers advantages over chemical methods by eliminating the need for synthetic surfactants and reducing toxicity. Biomolecules, such as peptides, amino acids, and secreted organic acids, form a biological coating around nanoparticles, influencing their surface properties and interactions with biological systems (Sidhu et al., 2022).

Understanding biosynthetic mechanisms paves the way for the rational design of BNPs with specific properties tailored to each agricultural challenge. Future research could focus on metabolic or genetic optimization to increase nanoparticle yield and control physicochemical properties. For example, upregulation of NADPH-dependent reductases or modulation of EPS secretion may improve nucleation control and particle uniformity.

### 2.1 Optimizing synthesis—the path to custom-made nanoparticles

In addition to optimizing culture conditions, selecting bacterial strains with high activity of specific reducing enzymes (e.g., nitrate reductase and quinone reductase) offers a promising route to improve BNP synthesis. Strains that abundantly produce exopolysaccharides (EPS) or biosurfactants can yield BNPs with enhanced stability and biocompatibility. The biological capping formed by these metabolites not only prevents nanoparticle aggregation but also facilitates interactions with plant tissues and microbial communities, acting as a molecular interface that influences the environmental behavior, fate and function of BNPs within the agroecosystem.

In the long term, metabolic engineering of microorganisms to overexpress genes involved in the production of reducing agents or to produce capping peptides with specific functionalities (e.g., antimicrobial peptides, nutrient-chelating molecules) could revolutionize the production of BNPs, including for specific agricultural challenges. For example, engineering bacteria to secrete siderophore-like peptides during nanoparticle synthesis could create iron BNPs with enhanced bioavailability for plants.

Although the application of genetically modified organisms in agriculture faces regulatory barriers and challenges to public acceptance, this approach represents a technological frontier that warrants attention from the scientific community and could unlock unprecedented control over nanoparticle properties and functions. While public and regulatory concerns are understandable, it is important to recognize that, compared with conventional pesticide use, the controlled use of engineered microorganisms may ultimately pose fewer ecological risks. Genetically optimized strains can be designed for self-limiting growth, targeted activity, and rapid environmental degradation, reducing persistence and bioaccumulation. In this context, microbial engineering should not be seen as a threat to biosafety, but rather as an opportunity to replace broad-spectrum chemical inputs with precision biological tools.

## 3 Integrated agricultural applications of biogenic nanoparticles

### 3.1 Phytosanitary action and pathogen control

The synthesized nanoparticles demonstrate exceptional antimicrobial properties against plant pathogens, as their mechanism of action is multifaceted and involves the generation of reactive oxygen species (ROS), disruption of cell membrane integrity, and interference with essential metabolic processes (Jha et al., 2025; Padmavathi and Satya Anuradha, 2022; Rupanshi et al., 2025). Biogenic silver nanoparticles are effective against a wide range of phytopathogens, including gram-positive and gram-negative bacteria, fungi, and certain viruses (Alfosea-Simón et al., 2025). Approximately 650 different types of microorganisms are susceptible to silver, making it a valuable tool for plant protection (Ahmed et al., 2016).

The mode of action involves the controlled release of silver ions, which interact with sulfhydryl (-SH) groups in essential pathogen proteins, leading to protein denaturation and cell death. Additionally, nanoparticles can directly penetrate pathogenic cells and induce DNA damage (Ashraf et al., 2025; Rodrigues et al., 2024).

The high surface-area-to-volume ratio of nanoparticles allows increased penetration and interaction with plant tissues, facilitating the targeted delivery of bioactive compounds and nutrients (Dutta et al., 2022; Li et al., 2023). Another advantage is that nanoparticles can be engineered to self-assemble into various structures, such as spherical shells, nanotubes, and sheets. These features enhance their multifunctionality and biodegradability, making them suitable for phyto-nanotechnological applications (Yu et al., 2022).

### 3.2 Function as nanofertilizers

In addition to their antimicrobial properties, BNPs act as an advanced nutrient delivery system that offers advantages over conventional fertilizers (Di Rago et al., 2025). The controlled release of ions enables more efficient absorption by plants, thereby reducing nutrient losses from leaching and volatilization (Arora P. K. et al., 2024; Dhage and Vidyashree, 2022; Goyal et al., 2023).

Nanoparticles can penetrate plant tissues through the stomata and cuticular pathways in leaves, as well as through root epidermal cells, ensuring efficient nutrient delivery (Bouyahya et al., 2022). This dual mechanism maximizes nutrient availability, promoting better growth, greater stress tolerance, and higher yields (Djanaguiraman et al., 2024; Maaz et al., 2025). Biologically synthesized calcium, magnesium, and silicon nanoparticles increase biomass, yield, and stress tolerance in legume crops (Singh et al., 2025).

The gradual release of iron, zinc, copper, and manganese from bacterial nanoparticles significantly influences the expression of root transporters in plants, affecting their absorption and homeostasis mechanisms. These micronutrients are essential for various physiological processes, including chlorophyll synthesis, photosynthesis, and respiration. Their absorption is strictly regulated by specific transporters, such as the ZIP (ZRT, IRT-like proteins) family. Metal transporters are crucial for acquiring metals from the soil. This process can improve the nutritional content of plants by accumulating essential metals and excluding toxic ones (Hussain et al., 2023; Sembada and Lenggoro, 2024; Wang et al., 2023).

### 3.3 Case studies and practical results

Emerging evidence suggests that BNPs can favor beneficial microorganisms while suppressing deleterious ones. This selectivity may arise from differences in cell wall composition, efflux pump expression, or biofilm formation capacity among microbial taxa.

Bacterial BNPs are effective in promoting plant growth and can act as nanofertilizers, pesticides, or a combination of both (Rupanshi et al., 2025). Field trials using silver, gold, zinc, copper, and manganese BNPs have demonstrated activity against more than 16 different pathogens, including bacteria, fungi, and viruses (Ali et al., 2024). In fava bean (*Vicia faba*) and guar bean (*Cyamopsis tetragonoloba*) crops, foliar application of silver BNPs from *Bacillus* sp. resulted in effective control of viral pathogens such as Bean Yellow Mosaic Virus (BYMV) and *Tobamovirus crotalariae*, respectively, leading to complete suppression of symptoms (Alfosea-Simón et al., 2025). Mittal et al. (2021) demonstrated that BNPs produced by plant growth-promoting bacteria enhanced plant resistance and promoted growth and overall plant health by acting as phytosanitary agents.

Silver BNPs from *Bacillus siamensis* improved rice seedling growth by inhibiting the pathogen *Xanthomonas oryzae* pv. *oryzae* (Ibrahim et al., 2019). Silver BNPs from *Bacillus cereus* control infection by *X. oryzae* pv. *oryzae* and exhibit antioxidant activity, providing protection to plants while reducing stress levels (Ahmed et al., 2020). Using silver BNPs synthesized from various organisms (*Pseudomonas* sp., *Achromobacter* sp., *Trichoderma* sp., and *Cephalosporium* sp.), Kaur et al. (2018) demonstrated improved chickpea germination by combating the pathogen *Fusarium oxysporum* f. sp. *ciceris*.

Some nanoparticles can be used to alleviate abiotic stress, such as temperature fluctuations, drought, salinity, and heavy metal contamination. Copper nanoparticles (CuNPs) from *Klebsiella pneumoniae* improve corn plant growth in high-salinity soil and promote antioxidant activity (Noman et al., 2021). Wheat plants treated with CuNPs synthesized from *K. pneumoniae* and *Shigella flexneri* showed reduced cadmium absorption in contaminated soils, improving nutrient uptake and enhancing plant growth (Noman et al., 2020a,b) Molybdenum nanoparticles from *Bacillus* sp., also used in wheat plants, reduced arsenic absorption by 30.3% (Ahmed et al., 2022).

Beyond their direct effects on plants and pathogens, bacterial BNPs possess a unique capacity to modulate the composition and function of plant-associated microbial communities. This property is rooted in the biological capping that envelops BNPs, composed of proteins, polysaccharides, and other bacterial metabolites. Unlike synthetic nanoparticles with chemical coatings, the biological corona of BNPs is recognized as “self” by microbial communities, reducing non-specific toxicity while enabling selective interactions (Quinteros et al., 2019).

Biogenic Silicon nanoparticles can improve microbial diversity and plant resistance by promoting beneficial symbiotic interactions (Shi et al., 2025). The result is a rebalanced microbiome that supports plant health through multiple mechanisms: enhanced nutrient solubilization, phytohormone production, induction of systemic resistance, and competitive exclusion of pathogens.

Furthermore, BNPs may serve as priming agents in seeds, preparing the plant-microbiome system for future challenges (Pereira et al., 2021). Application of BNPs during seed treatment or early growth stages could recruit and establish a beneficial microbial consortium in the rhizosphere from the outset, creating a foundation for long-term plant resilience. This concept of “microbiome engineering” via BNPs represents a paradigm shift from viewing nanoparticles as standalone inputs to recognizing them as tools for shaping the complex biological networks that underpin agricultural productivity and sustainability.

## 4 Perspectives and challenges

### 4.1 Regulation and risks

The prospects for using biogenic nanoparticles in agriculture open new pathways and encourage disruptive thinking. Modern agriculture offers numerous possibilities and innovative approaches to problem-solving, one of which is the application of nanotechnology, given the promising convergence between ecological nanotechnology and sustainable agriculture.

The unique ability of bacteria to produce functional nanoparticles under challenging environmental conditions positions this technology as a viable alternative to traditional synthetic methods. However, as with any innovation, challenges remain that must be addressed to foster progress in both agriculture and sustainability.

Since economic viability depends on factors such as raw material costs, conversion efficiency, and product added value, the use of agro-industrial waste as a substrate makes the process more competitive than synthetic methods (Abd-Elhalim et al., 2025; Meng et al., 2025). Therefore, bacterial synthesis, similar to other biogenic syntheses, offers opportunities to implement circular economy principles in agriculture, especially with the use of agricultural waste as a substrate for bacterial growth, creating value from discarded materials (Abd-Elhalim et al., 2025; Saxena et al., 2025).

From a sustainability perspective, BNPs offer clear advantages, including reduced use of synthetic agrochemicals, lower energy consumption in production, and greater compatibility with plant microbiomes. The possibility of using agro-industrial waste as a substrate further supports the circular economy dimension. BNPs also provide advantages over synthetic nanoparticles (Table 1).

**Table 1:** Comparison between biogenic and synthetic nanoparticles for agricultural applications.

Parameter	Biogenic nanoparticles	Synthetic nanoparticles
Synthesis Method	Enzymatic reduction by bacteria	Physical–chemical methods
Production Conditions	Room temperature, neutral pH	High temperatures, extreme conditions
Energy Consumption	Low (metabolic processes)	High (heating, pressure)
Environmental Impact	Minimal, biodegradable	Moderate to high, toxic residues
Biocompatibility	High (biological corona)	Variable (depending on coating)
Toxicity	Low (natural components)	Moderate to high (chemical residues)
Nutrient Release	Controlled and sustained	Fast, potential leaching
Interaction with Microbiome	Compatible, selective	Potentially disruptive
Scalability	Good (fermentation processes)	Excellent (industrial processes)
Sustainability	High (circular economy)	Low to moderate

The transition from laboratory-scale bacterial synthesis to industrial production may be one of the main challenges for commercialization, as it requires optimization of several parameters (Carmona et al., 2023; Narayanan and Sakthivel, 2010). However, fermentation processes are already established in the biotechnology industry, and large-scale microbial cultivation is a common practice.

Although BNPs represent a promising alternative to synthetic pesticides owing to their higher biocompatibility, long-term studies are still required to evaluate their environmental impacts, particularly regarding effects on non-target organisms, soil persistence, and potential bioaccumulation (Bouhadi et al., 2025; Mgadi et al., 2024).

Significant progress has been made in the regulatory landscape for agriculture, although considerable challenges remain. Different jurisdictions have adopted diverse approaches to safety assessment (Amenta et al., 2015; Rodríguez-Gómez et al., 2025). The European Union has established specific frameworks under the Registration, Evaluation, Authorization and Restriction of Chemicals (REACH) regulation, requiring detailed environmental and human risk assessments (Vinci et al., 2025). In the United States, the EPA, FDA, and USDA are collaborating to establish clear guidelines for agricultural nanotechnology products. Developing standardized testing protocols will be crucial for accelerating approval processes (Bouhadi et al., 2025; Rodríguez-Gómez et al., 2025).

The regulatory approval of BNPs for agricultural use requires not only demonstration of efficacy and safety but also detailed physicochemical characterization of the nanoparticles. The characterization of the biological capping is particularly important, as it is the defining feature of BNPs relative to synthetic nanoparticles and directly influences biocompatibility, stability, and interactions with biological systems. Regulatory frameworks specific to BNPs are still under development, and collaboration among the scientific community, industry, and regulatory bodies will be essential to establish guidelines that are both rigorous in terms of safety and technically and economically feasible.

### 4.2 The next frontier: a vision for integrated bionanotechnology

**Figure 2:**
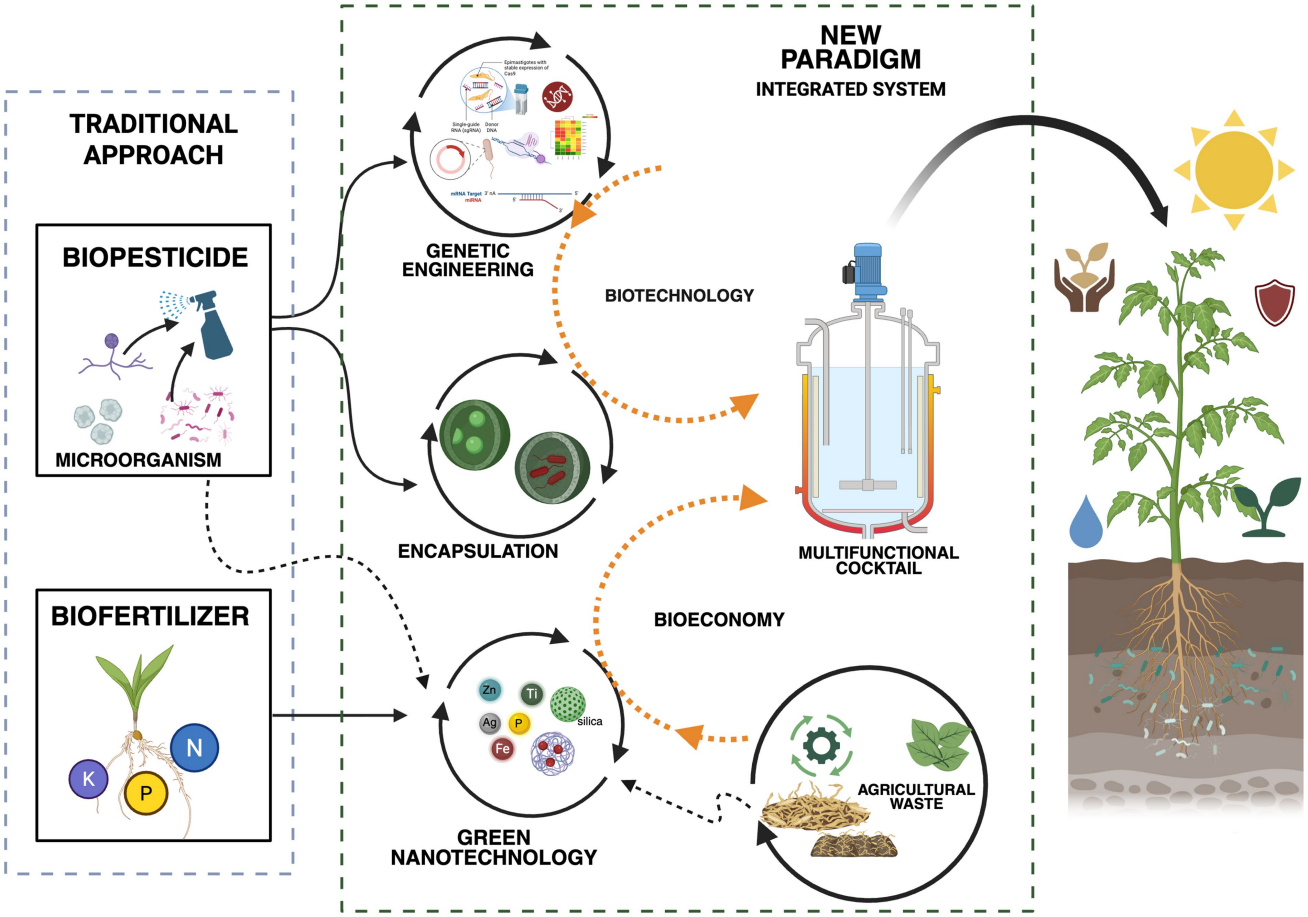
Integration of biotechnology and circular bioeconomy through bacterial biogenic nanoparticles for resilient agroecosystems.

The perspective of “Integrated BNP-Plant-Microbiome Systems” paves the way for a new generation of agricultural technologies that move beyond the isolated use of single inputs. We envision the development of multifunctional “cocktails” of bacterial nanoparticles, comprising BNPs of different metals synthesized by microbial consortia, strategically designed to simultaneously modulate multiple aspects of the agroecosystem (Figure 2). For instance, a formulation integrating silver BNPs (for biocontrol), iron and zinc BNPs (for nutrition), and silicon BNPs (for stress resilience) could provide an integrated, synergistic, broad-spectrum solution for crop management. The interactions among these nanoparticles, mediated by their distinct but complementary mechanisms of action, may yield emergent effects that surpass the sum of their individual contributions.

Beyond multi-nanoparticle formulations, the integration of BNPs with other bio-inputs holds immense promise. BNPs could be co-applied with bioinoculants to generate even more pronounced synergistic effects. For instance, BNPs could act as “priming agents” that prepare the plant-microbiome system to respond more effectively to biotic and abiotic challenges. Their use in seed treatments could not only provide nutrients and protection during early growth stages and during seed storage but also recruit and establish a beneficial rhizosphere microbiota from the very beginning of plant development, creating a foundation for long-term resilience.

In the long term, integrating BNPs with monitoring technologies such as soil sensors, satellite imagery and predictive models could enable truly autonomous and resilient agricultural systems, where input application is optimized in real time according to the crop needs and environmental conditions. This convergence of biogenic nanotechnology, microbiology, and data science represents the frontier of 21st-century sustainable agriculture. However, achieving this vision requires overcoming key challenges in scalability and formulation. Large-scale fermentation processes must be optimized for consistency and cost-efficiency, while formulations need to maintain the colloidal stability and biological activity of bacterial BNPs during storage and application. Addressing these challenges will demand strong interdisciplinary collaboration and sustained investment in research and innovation -but the potential rewards- a sustainable, productive, and resilient agricultural paradigm—are well worth the effort.

We envision a future in which bacterial nanofactories contribute directly to circular agricultural systems, coupling waste valorization with precision nutrient delivery and plant protection. By integrating microbial biotechnology with green nanotechnology, bacterial BNPs could redefine approaches to soil health, plant nutrition, and sustainable productivity.
